# Delivery through Shell Wound

**Published:** 1917-07

**Authors:** 


### ABSTRACTS

**Delivery Through Shell Wound.** Saint, Goelinger, and Poiré record (Journ. de méd. et de chir. prat., January 10, 1917) the case of a woman six months pregnant in whom delivery was brought about by a remarkable accident. She lived in a region occupied by the British and constantly bombarded, and was sitting at a window when a shell exploded in the street and wounded her in the lower abdomen. When brought to the hospital it was found that the belly was very painful and palpation was so difficult that it was impossible to determine the position of the fetus. An aperture of entry was found a little below and to the left of the umbilicus, and that of exit at a distance of nine centimetres from the left crural arch. On palpation it was found that the abdominal muscles were completely divided and that only a bridge of skin was left between the two apertures. The patient was bleeding abundantly through the vagina. The bridge of skin was cut through and laparotomy was performed. On the fundus there was found a wound of about five centimetres through which was seen the lumbar region of a fetus showing a small wound. The wound was enlarged, when the fetus was easily delivered; the pelvis, which was full of meconium and amniotic fluid, was cleaned, and the operation was completed by careful haemostasis and suture of the uterus.

The case ran a normal course and the mother made a rapid recovery. As for the child, which was left unattended to as it was believed to be dead, it soon began to cry and to show itself very much alive. It weighed 950 grams; but as no incubator was available it died in fifteen hours. The British Medical Journal, December 4, 1915, stated that Dr. Henrot had not long before given to the Paris Académie de Médecine an account of the bombardment of the hospital at Rheims. The maternity patients were by way of precaution moved to the cellars; one of the women was delivered by the action of a shell, which tore open the abdomen and uterus; the child had simply to be extracted.—(Brit. M. J., Feb. 17, 1917.) Med. Times, June.

